# Landowner Perceptions of Heronry Conservation in Human‐Dominated Wetlands of Bangladesh

**DOI:** 10.1002/ece3.73938

**Published:** 2026-07-01

**Authors:** Allama Shibli Sadik, Muntasir Akash, Ashis Kumar Datta, Samiul Mohsanin, Md. Sher‐E‐Afgan, Rima Akter, Humayun Kabir

**Affiliations:** ^1^ Wildlife Center, Forest Department Gazipur Bangladesh; ^2^ Department of Zoology Jahangirnagar University Dhaka Bangladesh; ^3^ Forest Department Dhaka Bangladesh; ^4^ Department of Zoology University of Dhaka Dhaka Bangladesh; ^5^ Wildlife Conservation Society Bangladesh Dhaka Bangladesh; ^6^ Prokriti O Jibon Foundation Dhaka Bangladesh

**Keywords:** Anhingidae, Ardeidae, Ciconiidae, colonial waterbirds, community perceptions, landowner tolerance, Phalacrocoracidae, wetland conservation, wetland‐dependent species

## Abstract

Heronries—tree‐nesting colonies of waterbirds—are globally threatened by wetland loss, yet their conservation remains poorly understood. In Bangladesh, heronries occur in shared wetland‐agricultural landscapes, making landowner tolerance a proximate determinant of colony persistence. However, socially informed research on heronry conservation is entirely absent in Bangladesh and rare globally. We conducted focus group discussions at 235 heronry sites across northern Bangladesh, engaging 2555 participants. Drawing on Social‐Ecological Systems theory, we examined the human‐heronry interface: landowner motivations for tolerance, threats, problems experienced, and preferred conservation interventions. We quantified threat perceptions and identified structural patterns in motivations, problems, and conservation preferences. Habitat loss and poisoning emerged as the most severe threats, with poisoning exceeding 60% weighted severity in all areas. Landowner tolerance was high and primarily driven by ethical concern, environmental benefits, and social recognition. Pattern analysis revealed significant clustering of motivations, problems, and conservation preferences, indicating non‐random and interpretable socio‐ecological structure. Strong stench from guano fouling was the most widespread problem, while habitat protection, awareness programmes, and signage were the most co‐preferred conservation interventions. Financial compensation was supported at 211 of 235 sites, with annual demands averaging USD 348.0 ± 311.6 per site—figures that reflect perceived costs of hosting colonies. Heronry conservation is a structured socio‐ecological challenge amenable to targeted, multi‐pronged interventions. Our findings demonstrate that least concern species require locally tailored social strategies for persistence in shared landscapes. Three transferable principles emerge for heronry conservation in human‐dominated wetlands: (i) landowner tolerance is multifactorial and fragile—currently high but vulnerable to erosion where problems go unaddressed—and must be engaged through simultaneous ethical, environmental, social, and economic pathways; (ii) threat severity is spatially heterogeneous, requiring site‐specific adaptive management in the absence of effective enforcement even within formally recognised conservation areas; and (iii) pattern‐based diagnostics of community survey data offer a scalable and accessible tool for identifying conservation leverage points in data‐limited systems.

## Graphical abstract

Heronries—tree‐nesting waterbird colonies—face growing threats in Bangladesh's shared wetland landscapes, where landowner tolerance is the proximate determinant of colony persistence. Through focus group discussions at 235 heronry sites with 2555 participants, we found tolerance to be high but multifactorial, driven by ethical concern, environmental benefits, and social recognition, while habitat loss and poisoning emerged as the most severe threats with marked spatial heterogeneity. Network analysis revealed modular clustering in motivations, problems, and conservation preferences, demonstrating that heronry conservation is a structured socio‐ecological challenge requiring simultaneous ethical, environmental, social, and economic interventions tailored to local conditions.

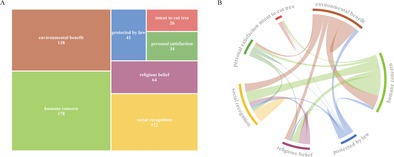

## 1 Introduction

Heronries are the spatio‐temporal congregations of waterbirds that are ecologically dependent on wetlands (Ramsar Convention 1994) and that breed and roost in trees (Roshnath 2014; Roshnath and Sinu 2023). In Bangladesh, heronries are mixed colonies, primarily composed of families, such as Ardeidae (herons and egrets), Ciconiidae (storks), Phalacrocoracidae (cormorants), and Anhingidae (darters), with occasional additions of roosting flocks of Threskiornidae (ibises) (Sadik et al. 2025). As wetland ecosystems face accelerating degradation (Convention on Wetlands 2021), heronries serve as indicators of wetland health, particularly in the Global South where anthropogenic footprints on freshwater ecosystems are most severe (Roshnath and Sinu 2023). Yet systematic research integrating ecological and social dimensions of heronry conservation remains limited worldwide and entirely absent in Bangladesh (Roshnath 2014; Roshnath and Sinu 2023)—a critical gap given that conservation outcomes in human‐dominated landscapes are fundamentally shaped by human behaviour, perceptions, and institutional responses (Roshnath and Sinu 2023).

Globally, 35% of natural wetlands are lost between 1970 and 2015 while heavy human use of wetlands has increased by more than twofold (Convention on Wetlands 2021). Bangladesh exemplifies this trajectory acutely. Wetlands, once comprising 80,000 sq. km in the 1970s, are now significantly damaged or degraded due to agricultural and farming conversions, flood control and damming projects, and other anthropogenic usages (Islam and Kitazawa 2013). Key freshwater wetland habitats in Bangladesh are at risk; for example, the Tanguar Haor, a Ramsar Site, has experienced more than 40% reduction of its original size (Haque and Basak 2017). The Hakaluki Haor, an Important Bird Area, has lost 50% of its fish diversity with increasing anthropogenic usages (Aziz et al. 2021). Almost the entirety of wetlands of the Ganges‐Brahmaputra confluence floodplains are now converted (Islam and Kitazawa 2013) with the region becoming highly drought‐prone (Paul and Pal 2020). Cascading declines in freshwater wetland ecosystems have driven the extirpation or near‐extirpation of several tree‐nesting colonial waterbird species in Bangladesh, including the greater adjutant ( *Leptoptilos dubius* ), lesser adjutant ( *Leptoptilos javanicus* ), and oriental darter ( *Anhinga melanogaster* ) (see IUCN Bangladesh 2015; Sadik et al. 2024).

Heronries, unlike many sea‐faring colonial waterbird families, such as Laridae (gulls), Diomedeidae (albatrosses), and Sturnidae (terns), do not garner adequate conservation focus (Fasola and Alieri 1992; Roshnath and Sashikumar 2019). Nevertheless, declines in heronries are increasingly acknowledged worldwide (see Fasola and Alieri 1992; Roshnath and Sashikumar 2019; Byju et al. 2025). Systematic monitoring of mixed heronries in the agricultural landscapes of the Po Valley, southern Europe, conducted since 1982, has demonstrated that long‐term colony‐level tracking is essential for detecting population trends and informing adaptive management (Fasola and Alieri 1992; Hafner and Fasola 1997). In southern India, heronry conservation has received significant traction, with studies in Tamil Nadu finding that vegetation structure and urban tree characteristics influence nest site selection (Frank et al. 2021), and work in Kerala demonstrating that cultural beliefs, religious values, and community engagement are central determinants of landowner tolerance and colony persistence (Roshnath 2014; Roshnath, Athira, et al. 2019; Roshnath, Sashikumar, et al. 2019; Roshnath and Sinu 2023).

Furthermore, contrary to seabird colonies that often occupy remote or legally protected areas, heronries predominantly occur in privately owned homestead trees within densely populated agricultural and peri‐urban landscapes (Sadik et al. 2025). This spatial configuration creates a distinctive conservation challenge: the persistence of colonies is determined not primarily by formal protected area governance, but by the day‐to‐day decisions of individual landowners who simultaneously bear the costs of hosting colonies and retain full authority over nesting trees. This dynamic is well‐captured by social‐ecological systems (SES) theory (Ostrom 2009), which emphasises that conservation outcomes emerge from interactions between ecological units (e.g., wildlife populations or habitat patches), resource users, governance systems, and broader social‐ecological contexts. Heronries in Bangladesh constitute precisely such SES units—ecologically defined by wetland dependency, socially embedded in private landholdings, and institutionally underserved by existing conservation frameworks. Within this SES lens, landowner tolerance—defined here as the willingness to permit continued colony presence despite associated costs (e.g., guano fouling, crop damage, or loss of nesting trees)—emerges as a proximate determinant of colony persistence. Tolerance in human‐wildlife systems is rarely unidimensional; it is shaped by motivational clusters encompassing ethical concern, perceived ecological and economic benefits, cultural and religious values, and social norms (Kansky et al. 2016; Nyhus 2016). Understanding the structure of these motivational clusters, and how they interact with experienced problems and conservation preferences, is essential for designing realistic, locally appropriate interventions. Yet the motivational architecture of heronry tolerance has never been examined, particularly in the context of densely populated South Asian wetland landscapes.

Systematic monitoring and conservation concern on heronries in Bangladesh has been evolving since 2020 (see Aziz 2023; Sadik and Akash 2024; Sadik et al. 2025). These efforts provide important insights into the distribution and breeding biology of tree‐nesting colonial waterbirds, such as little cormorants ( *Microcarbo niger* ), great egrets ( *Ardea alba* ), and black‐crowned night herons (see Naher et al. 2009; Reza et al. 2010; Naher 2014; Kabir et al. 2019). Previously unknown breeding accounts of large storks have been documented, such as lesser adjutants (Sadik et al. 2024) and Asian woollynecks ( *Ciconia episcopus* ) (Hasan and Ghimire 2020), both critically endangered in the country. With the conservation momentum on heronries, conservation challenges are also recognised, which are largely associated with shrinking habitats and increasing anthropogenic dependency on wetlands (Sadik et al. 2024; Sadik et al. 2025)—bringing heronries into close contact with humans, generally perceived as a nuisance (Roshnath and Sashikumar 2019; Hasan and Ghimire 2020; Sadik et al. 2024; Sadik et al. 2025). Furthermore, habitat loss and direct persecution—through poisoning, hunting, and collecting eggs or chicks for consumption or trade—are globally recognised pressures on colonial waterbird populations (Kushlan and Hafner 2000; Erwin et al. 2003; Roshnath and Sashikumar 2019). However, the relative severity of these threats varies substantially across spatial and social contexts, and landowner perceptions of threat severity do not always align with ecological assessments (Dickman 2010). Understanding perceived threats from the landowner perspective is therefore critical—not only for ecological monitoring but for identifying entry points for conservation communication and, where needed, behaviour change.

To address these gaps, we draw on focus group discussions (FGDs) conducted across all 235 active heronry sites identified in northern Bangladesh (Sadik et al. 2025) to examine the human dimensions of heronry conservation through an SES lens. We recruited landowners as our primary study population because they manage the trees where colonies occur, experience most heronry‐associated costs, and ultimately determine whether colonies persist. We employed weighted network analysis to reveal non‐random structural patterns in social responses—a methodological approach that moves beyond descriptive reporting to identify conservation leverage points. Specifically, we address four interrelated questions: (i) Why do landowners tolerate heronries, and are their motivations structured non‐randomly? (ii) How severe and spatially heterogeneous are perceived human‐induced threats? (iii) What problems do landowners associate with heronries, and do these problems cluster predictably? (iv) What conservation interventions do landowners prefer, and do these preferences reflect coherent, integrative management strategies? By combining large‐scale social survey data with network‐based analytical approaches across a comprehensive site census, this study advances understanding of the socio‐ecological dynamics governing heronry persistence in human‐dominated wetland landscapes, with transferable implications for colonial waterbird conservation and wetland management across the Global South.

## 2 Methods

### 2.1 Study Area

**FIGURE 1:**
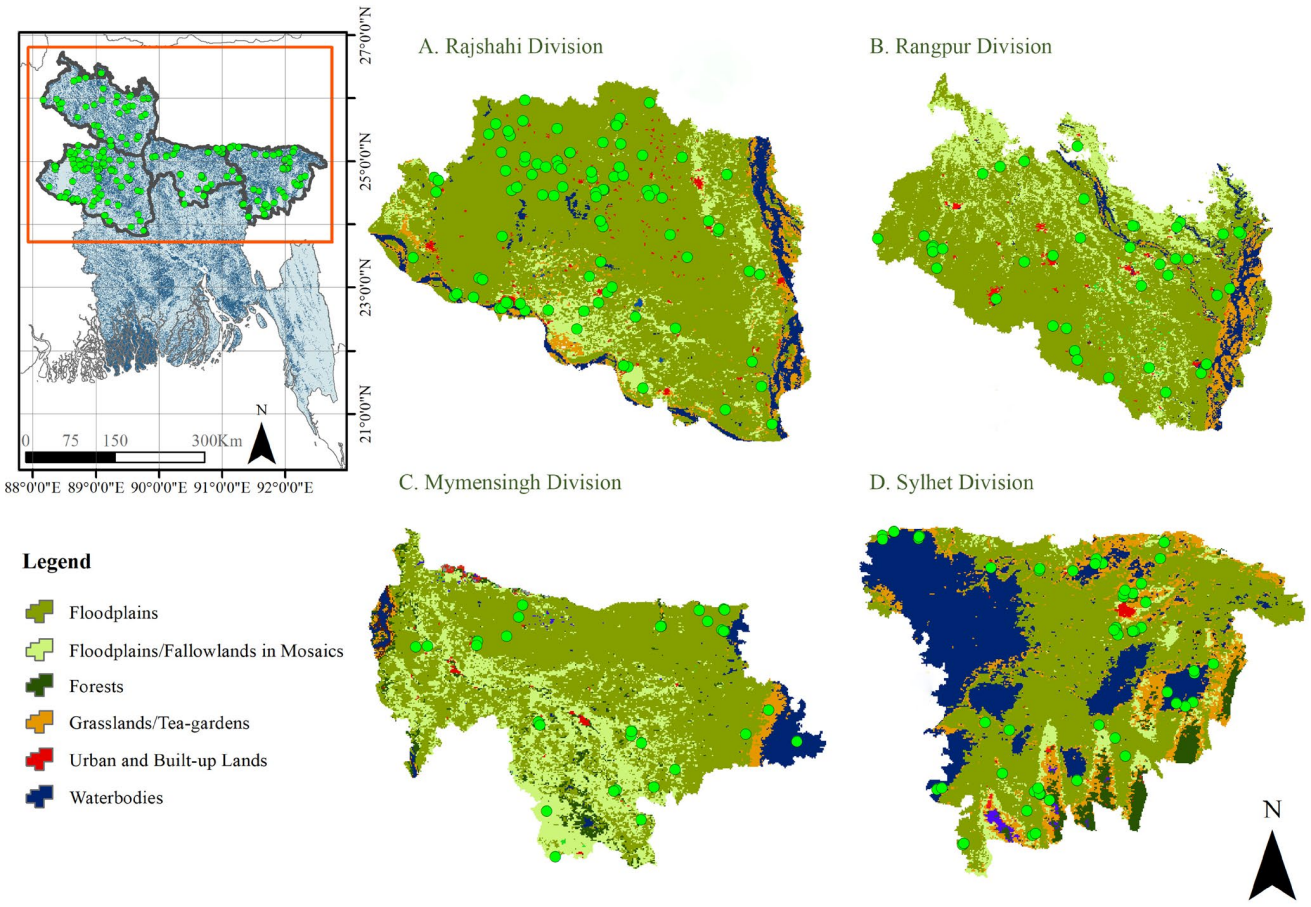
Study area showing heronry sites (green circles) across four administrative divisions in northern Bangladesh. The extent of land‐cover was sourced from the MODIS MCD12Q1 (v061) global 500 m raster dataset (Friedl and Sulla‐Menashe 2019), and wetland (inset) from the CIFOR global 232 m raster dataset (Gumbricht et al. 2017).

We carried out the study in four of the six divisions (first‐order administrative units) in northern and northeastern Bangladesh (Figure 1). The area encompasses extensive wetland–agricultural mosaics, riverine floodplains, and patchy secondary forests (Sadik et al. 2025). Several globally recognised wetlands and inland depressions of importance occur in the region, including two Ramsar sites—Tanguar Haor and Hakaluki Haor—and eight Important Bird Areas (IBAs), such as the Brahmaputra River and Hail Haor (BirdLife International 2026). This region was selected because it supports the highest documented density of active heronries in Bangladesh and represents a gradient of human population densities, land‐use intensities, and wetland conditions, making it well‐suited for examining variation in social responses to heronries across socio‐ecological contexts.

The four divisions in this region differ in their dominant wetland types, land‐use configurations, and livelihood systems, which collectively shape the socio‐ecological context of heronry conservation in the region. Sylhet and Mymensingh are characterised by expansive haor systems—seasonally inundated floodplain basins—where fishery‐dependent livelihoods predominate, and wetland connectivity remains relatively higher than elsewhere in the study area (Figure 1, BirdLife International 2026). Rangpur is dominated by riverine floodplains and char landscapes along the Teesta and Brahmaputra river systems, with agriculture and livestock grazing as primary livelihoods (Figure 1). Rajshahi, by contrast, encompasses drier floodplain mosaics along the Padma River system, where wetland coverage is comparatively reduced, and agricultural intensification is more pronounced (Figure 1). Human population densities around the heronry sites are highest in Rajshahi and lowest in the haor‐dominated areas of Mymensingh, broadly consistent with the household density patterns observed near heronry sites (Sadik et al. 2025). In terms of conservation governance, Sylhet hosts both Ramsar Sites and several IBAs positioned along the East Asian‐Australasian Flyway, bringing comparatively greater institutional conservation attention to the division. Mymensingh and Rangpur contain additional IBAs, including the Brahmaputra River and Hail Haor, whereas Rajshahi has relatively no internationally designated conservation areas within the study footprint (BirdLife International 2026).

### 2.2 Heronry Database and Site Selection

We targeted all 235 active nesting heronry sites identified by Sadik et al. (2025), ensuring complete spatial coverage rather than a sample. This census‐based design is a key strength of the study: rather than selecting a subset of sites, we conducted social surveys at every active heronry in the study area, eliminating spatial sampling bias and enabling robust division‐level comparisons. The heronry database was compiled between 2017 and 2024 using a composite methodology integrating citizen‐science reports, media records, global databases (eBird), and local youth conservation networks, with all sites verified through direct field visits (Sadik et al. 2025). At the end of the survey, 235 active nesting colonies were recorded, comprising 171,552 individuals of 16 species spread across 24 of 33 districts in the study area. For full methodological details and ecological results, refer to Sadik et al. (2025).

### 2.3 Focus Group Discussions

We selected FGDs as our primary data collection method for three reasons. First, heronries are spatially embedded in social communities where attitudes are shaped by collective norms and shared experiences; FGDs capture this social dimension more effectively than individual interviews (Kitzinger 1995). Second, FGDs are logistically appropriate for repeated deployment across 235 geographically dispersed sites with limited infrastructure access. Third, group deliberation in FGDs tends to surface locally dominant views and community‐level reasoning patterns—precisely the social dynamics that shape landowner behaviour toward heronries. With a standardised protocol, between 2022 and 2024, we carried out FGDs with the landowners based within 500 m of each heronry site—the stakeholders most directly affected by and interacting with these colonies (Table S1).

Of four research questions, landowners' reasons for tolerance and opinion on direct human‐induced threats to heronry were structured, with seven and four categories respectively, which are defined based on literature and field experience (Table 1). Additionally, each human‐induced threat category was further divided based on the reported intensity of occurrences: low (1%–25% of respondents reporting the threat), medium (26%–50%), high (51%–75%), and very high (76%–100%). We asked open‐ended questions on the human problems associated with heronry sites and possible conservation measures to overcome those possible challenges. Finally, we asked for landowners' opinion on yearly governmental monetary compensation for the protection of heronries on private lands.

**TABLE 1:** Definitions of standardised response categories used in coding FGDs.

*Landowners' reason of tolerance*	
Environmental benefit	Heronries provide ecological services (pest control, nutrient enrichment, maintaining local biodiversity, etc.)
Humane concern	Compassion toward birds, a belief that harming them is morally wrong, or a general desire to avoid killing wildlife
Intent to cut tree	Intend to remove nesting tree(s), but have postponed or avoided doing so for practical or social reasons (cost, community pressure)
Personal satisfaction	Derive personal pleasure, aesthetic enjoyment, or a sense of pride from hosting nesting birds
Protected by law	Awareness that birds or nesting colonies are legally protected, leading landowners to tolerate or avoid disturbing them
Religious belief	Tolerance grounded in religious values, teachings, or norms that discourage harming birds or promote coexistence
Social recognition	Hosting a heronry brings social prestige, respect, or positive recognition within the community
*Landowners' opinion on human‐induced threats*	
Habitat loss	Removal or degradation of nesting trees or associated habitat due to land‐use change, logging, or infrastructure development
Hunting	Intentional killing or capturing of birds for food, trade, or recreation
Poisoning	Use of toxic substances (often rodenticides or pesticides) that unintentionally or intentionally harm heronry birds
Stealing	Collection of eggs, chicks, or fledglings for consumption, trade, or keeping as pets
*Landowners' opinion on human problems associated with heronry sites*	
Strong stench	Complaints about foul smell from droppings or accumulated organic matter beneath the heronry
Movement difficulty	Physical inconvenience or obstruction (colonies positioned over entryways, pathways, courtyards, or work areas, etc.), which makes it difficult for landowners or household members to move freely around their homestead
Farming damage	Perceived negative impacts on crops (e.g., droppings, birds feeding in fields, contamination)
Roof damage	Damage to roofs of houses or structures due to droppings, nesting material, or water accumulation caused by blocked drainage
No problem	Landowners report no notable nuisance or negative impact from the presence of the heronry
*Landowners' opinion on possible conservation measures*	
Awareness protection	Increasing community awareness through education, meetings, or outreach to promote the protection of heronries
Community protection	Local community members collectively monitoring or safeguarding colonies through informal or organised efforts
Habitat protection	Planting, maintaining, or safeguarding nesting trees or associated habitat from degradation or removal
Law enforcement	Implementation or strengthening of legal measures to prevent hunting, egg collection, or habitat destruction
Rescue centre	Establishing or engaging wildlife rescue facilities to handle injured, fallen, or distressed chicks and adults
Sign boards	Installing informational or warning signage at heronry sites to encourage protection and reduce disturbance
Sheds	Simple sheds or covers beneath nesting trees to reduce droppings on roofs, courtyards, or walkways and minimise conflict

We documented FGDs through detailed notetaking rather than audio recordings. Each FGD was facilitated by a team of three to four trained coordinators and coded by a single trained coordinator following the standardised categories (Table 1). We did not include individual‐level sociodemographic data—including gender, age, educational level, and occupation—in this study, as our FGD format was designed to capture group‐level conservation perceptions rather than individual attributes. Informed verbal consent was obtained from all participants prior to each discussion. Confidentiality was strictly maintained, and no personally identifiable information is reported in this study.

### 2.4 Analytical Approaches

We calculated descriptive statistics to summarise patterns across the four research questions. To quantify the severity of human‐induced threats at the division level, we estimated a weighted severity index that accounts for both the presence and intensity of each threat. For every threat type (Table 1), we assigned numeric values to ordered severity levels: Absent = 0, Very Low = 1, Low = 2, High = 3, and Very High = 4, where 4 represents the maximum possible severity because it corresponds to the highest category in our ordinal scale. Weighted severity for each division and threat was calculated by summing these numeric values across all colonies within that division, such that higher scores indicate either more colonies being affected, higher intensity of the threat, or both. To facilitate interpretation and allow comparisons across divisions with different numbers of colonies, we additionally expressed weighted severity as a percentage of the maximum possible severity for that division using the formula:

$$\text{Weighted Severity}\;\left(\%\right)=\left(\frac{\sum \text{severity scores}}{\text{number of colonies}\times 4}\right)\times 100$$

where the denominator reflects the maximum total score that would occur if all colonies in the division experienced the highest severity.

Network analysis constitutes a central analytical contribution of this study. Rather than treating each motivational response, problem, or conservation preference as independent, we built undirected weighted networks from pairwise co‐occurrence matrices to reveal the structural organisation of social responses. In these networks, nodes represent individual responses and edge weights reflect co‐occurrence frequency across sites. This approach moves beyond standard frequency reporting to reveal whether certain motivations, problems, or conservation preferences are systematically associated—information that is critical for designing synergistic, multi‐lever conservation interventions (Barnes et al. 2017; Guerrero et al. 2020). We built undirected and weighted network structures to visualise the association between different responses to our queries on landowners' perception, human problems associated with heronry sites and possible conservation measures. We extracted all possible pairwise combinations and did not consider threshold for low intensity of co‐occurring response pairs to preserve all observed data. To test the non‐random modularity pattern in the observed network structure, we used the *bipartite* package (Dormann et al. 2008) in R (R Core Team 2025). We assessed the observed modularity with the Vázquez null model (see Vázquez et al. 2007), while preserving marginal totals of the observed matrix but randomising link distributions and tested significance with a one‐tailed test between the observed and the null modularity (alpha level 0.05). All analyses were conducted in R (R Core Team 2025) and ArcGIS, using WGS 1984 as the geographic datum.

## 3 Results

Across the 235 active heronries included in the study, heronry distribution varied among the four surveyed divisions (Table 2, Figure 1). Overall, this study engaged 2555 landowners as FGD participants across these sites, with an average of 10.87 (SD ± 3.84) participants per heronry site, reflecting consistent stakeholder engagement across the study area. However, the number of households residing within ≤ 500 m of heronry sites varied widely (Table 2, Figure 1), ranging from 01 to 230 per site, with a mean of 9.22 ± 20.42 families per heronry sites across the entire study area. Rajshahi exhibited the highest household densities near heronries, while Mymensingh had the lowest, indicating considerable spatial heterogeneity in human presence.

**TABLE 2:** Summary of heronry sites, focus group discussion participant numbers, and nearby household densities (≤ 500 m) across four divisions in northern Bangladesh.

Division	Heronries	Participants			Families		
		Total	Mean	SD	Total	Mean	SD
Mymensingh	32	300	9.38	2.15	134	4.19	3.25
Rajshahi	83	889	10.7	4.19	1241	15.0	32.9
Rangpur	59	648	11.0	3.85	462	7.83	6.12
Sylhet	61	718	11.8	3.85	332	5.44	5.76

### 3.1 Opinion on Human‐Induced Threats

Human‐induced threats were recorded in nearly all heronry sites. Only three colonies (1.3%) were free from all four human‐induced threats (habitat loss, hunting, poisoning, and stealing; see Table 1). Specifically, 19 heronry sites (8.1%) experienced at least one threat, 83 sites (35.3%) had at least two threats, 68 sites (28.9%) had at least three threats, and 62 sites (26.4%) were affected by all four threats.

**FIGURE 2:**
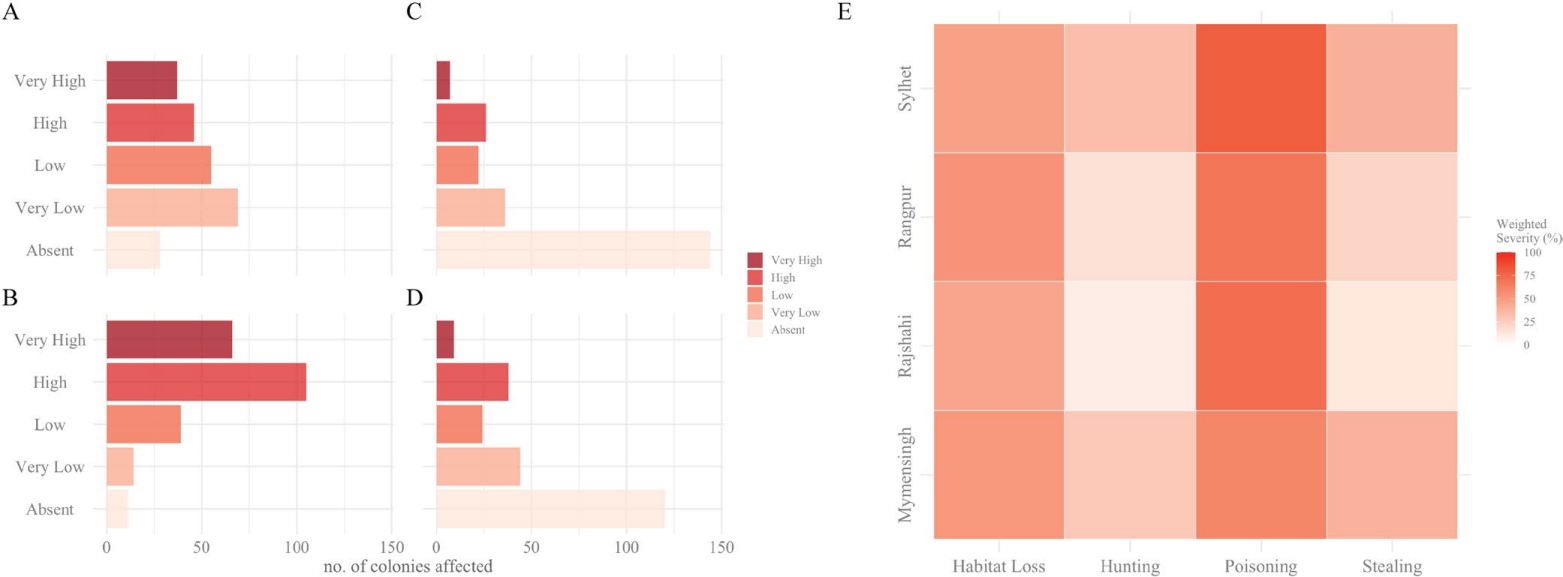
Human‐induced threats to heronry sites across northern Bangladesh. Bar charts showing the distribution of severity levels (absent, very low, low, high, very high) for four major threats: (A) habitat loss, (B) hunting, (C) poisoning, and (D) stealing. (E) Heatmap illustrating division‐wise weighted severity scores for each threat, standardised by the total number of heronry sites per division.

Severity of each threat, pooled across all sites and based on landowner perceptions, indicated that habitat loss and poisoning posed the most significant risks (Figure 2A–D). For habitat loss, 28 heronries experienced no threat, 69 very low, 55 low, 46 high, and 37 very high severity (Figure 2A). Hunting was largely absent in 144 heronries, with 36 very low, 22 low, 26 high, and 7 very high (Figure 2B). Poisoning was largely concentrated in the high severity categories, affecting 105 heronries (high) and 66 colonies (very high) (Figure 2C). Severity of collecting eggs and chicks was mostly absent or low, with 120 sites free from this threat, 44 very low, 24 low, 38 high, and 9 very high (Figure 2D).

The frequency of threat occurrence varied among divisions. Habitat loss was the most widespread, affecting 29 heronries in Mymensingh, 69 in Rajshahi, 52 in Rangpur and 57 in Sylhet. Poisoning was also common, notably in Rajshahi (*n* = 77), Rangpur (*n* = 55) and every heronry site in Sylhet. Stealing occurred in 23–43 colonies per division, while hunting was comparatively lower, ranging from 15 in Rajshahi to 33 in Sylhet.

Considering weighted severity, Figure 2E exhibited a distinct pattern. Poisoning scored > 60% for every division: Sylhet (78.7%), Rajshahi (71.9%), Rangpur (68.6%) and Mymensingh (60.9%). Habitat Loss was most severe in Rangpur (54.2%), followed by Sylhet (47.5%) and Rajshahi (46.4%). Stealing and hunting were comparatively less severe across divisions, with the highest weighted severity observed in Sylhet (40.2% and 33.2%, respectively).

### 3.2 Reason for Heronry Tolerance

**FIGURE 3:**
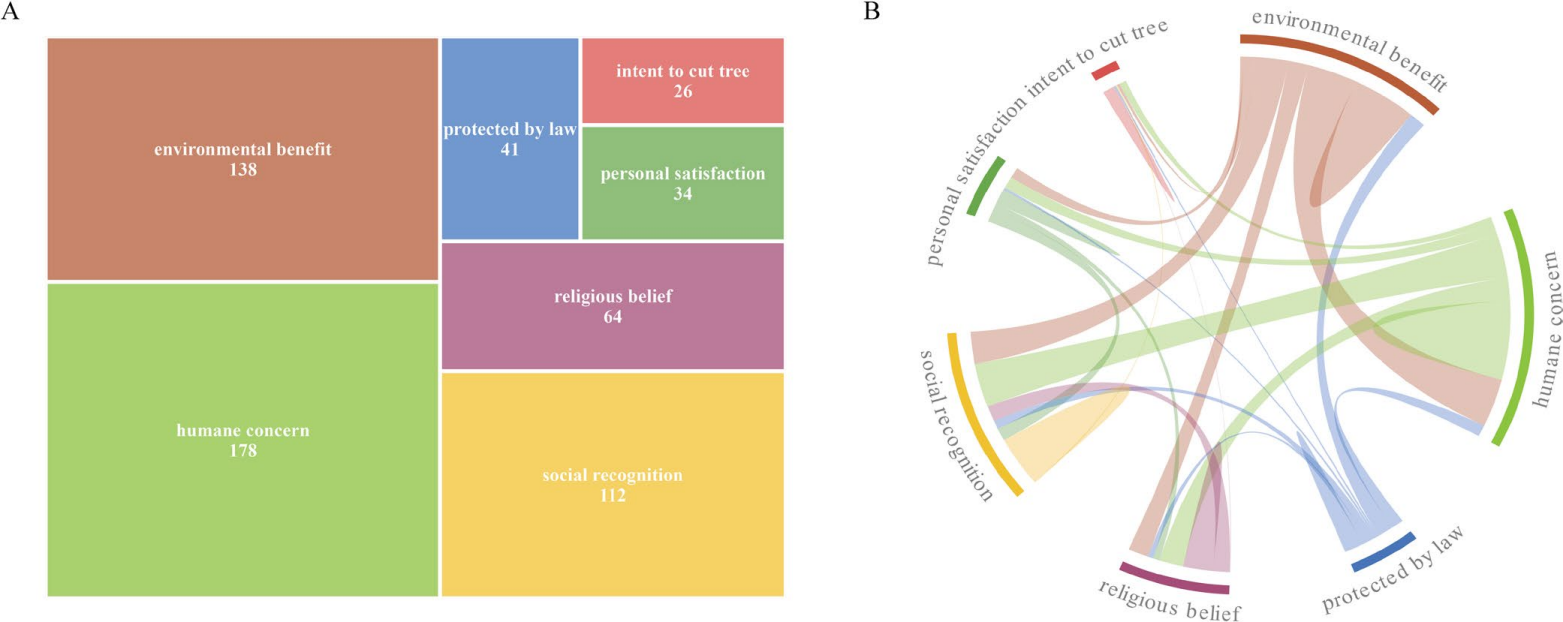
Reasons for heronry tolerance among landowners in northern Bangladesh. (A) Treemap showing the frequency of seven reported motivations. The size of each compartment is proportional to the number of times a motivation was cited, and numeric labels indicate those frequencies. (B) Chord diagram illustrating co‐occurrence of reasons, with arc widths proportional to frequency and connecting chords representing the number of co‐occurrences between pairs of motivations.

Landowners reported multiple motivations for tolerating heronries, with humane concern (*n* = 178), environmental benefit (*n* = 138), and social recognition (*n* = 112) being the most frequently expressed reasons (Figure 3A). Other motivations included religious belief (*n* = 64), protection under law (*n* = 41), personal satisfaction (*n* = 34), and a smaller fraction citing intent to cut trees (*n* = 26).

Unlike the other six categories, intent to cut trees does not reflect a positive motivation for hosting heronries; rather, it captures the conditional tolerance of landowners who are currently coexisting with colonies despite a preference for their removal, making it a conceptually distinct indicator of latent conflict risk.

Analysis of co‐occurring reasons revealed that ethical and environmental motivations are centrally connected (Figure 3B). For example, environmental benefit frequently co‐occurred with humane concern (*n* = 109) and social recognition (*n* = 73). Humane concern also overlapped with social recognition (*n* = 98) and protection under law (*n* = 26). In contrast, intent to cut trees appeared to be an isolated motivation, showing minimal connections with other reasons. The undirected, weighted network of provided responses exhibited significant modularity (*Q* = 0.31, *p* < 0.0001), supporting the observed motivation clusters.

### 3.3 Human Problems Associated With Heronry Sites

**FIGURE 4:**
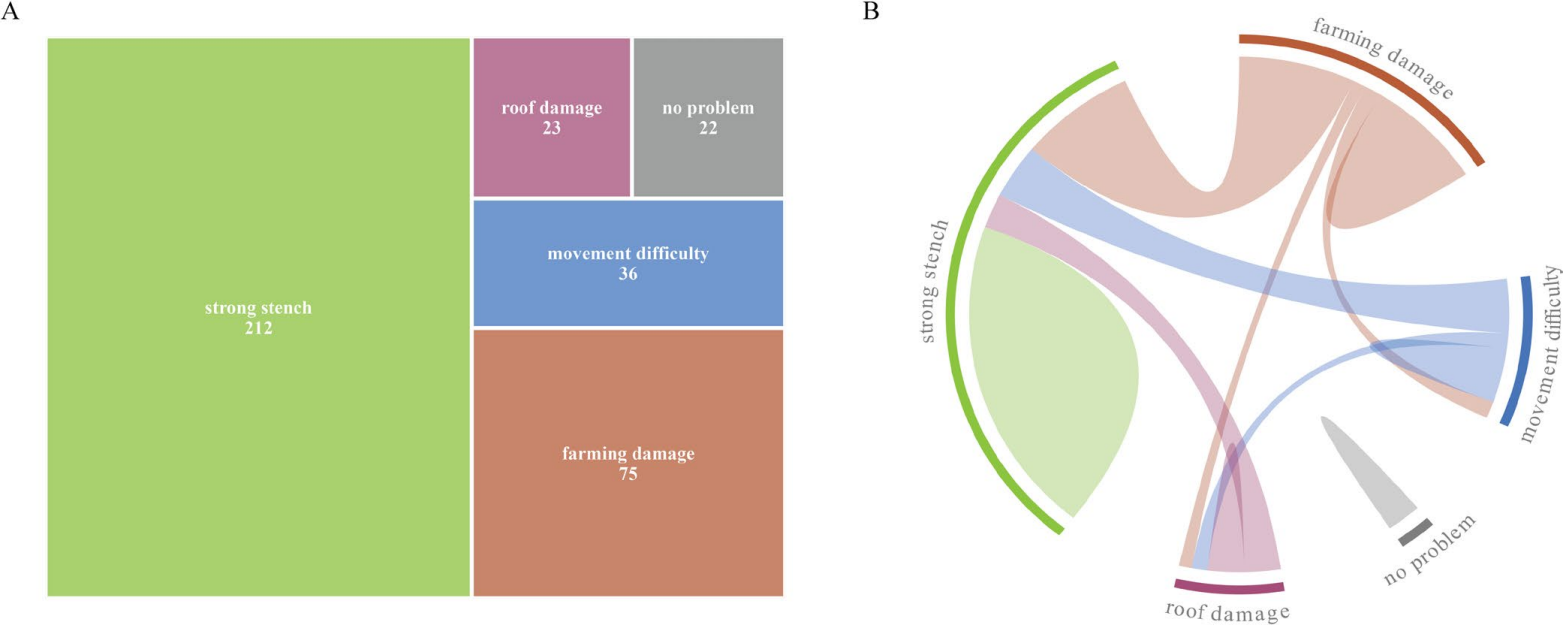
Human problems associated with heronry sites in northern Bangladesh. (A) Treemap showing the frequency of reported issues. The size of each compartment is proportional to the number of times a problem was reported, with numeric labels indicating those frequencies. (B) Chord diagram illustrating co‐occurrence of problems, with arc widths proportional to the frequency of each problem and connecting chords representing the number of times two problems were reported together.

Landowners reported four problems associated with heronry sites, with strong stench being the most frequently cited issue (*n* = 138), followed by farming damage (*n* = 75), movement difficulty (*n* = 25), roof damage (*n* = 15). At 22 heronry sites, respondents commented having no issue (Figure 4A).

Figure 4B indicates that while some problems overlap, the majority experienced strong stench and farming damage as a single, distinct issue. To a lesser extent, movement difficulty was reported alongside strong stench (*n* = 25) and roof damage (*n* = 6). The undirected, weighted network of human‐reported problems showed clear structural clustering (*Q* = 0.54, *p* = 0.01), suggesting that landowners tend to experience and report certain problems rather than providing random responses.

### 3.4 Perception on Possible Conservation Measures

**FIGURE 5:**
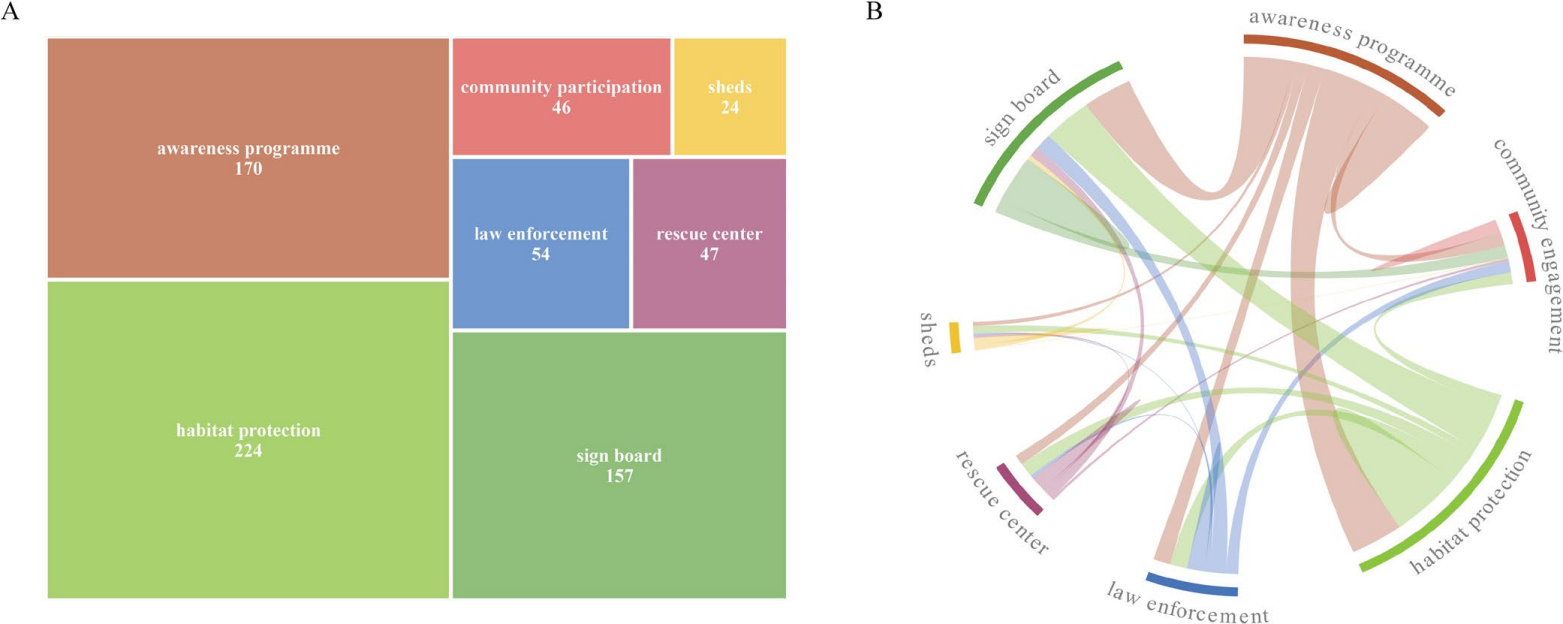
Perceived conservation measures for heronry sites proposed by landowners in northern Bangladesh. (A) Treemap showing the frequency of seven proposed conservation measures. The size of each compartment is proportional to the number of times a measure was proposed, and numeric labels indicate those frequencies. (B) Chord diagram illustrating co‐occurrence of proposed measures, with arc widths proportional to frequency and connecting chords representing the number of times two measures were proposed together.

FGD participants proposed seven different conservation measures for safeguarding heronry sites (Figure 5A). Habitat protection emerged as the most recommended action (*n* = 224), followed by awareness programmes (*n* = 170) and sign boards (*n* = 157). Other suggested interventions included community engagement (*n* = 46), law enforcement (*n* = 54), rescue centres (*n* = 47), and, less frequently, the installation of protective sheds beneath nesting trees (*n* = 24; see Table 1).

Patterns of co‐selection among proposed measures revealed notable associations (Figure 5B). For instance, awareness programmes were frequently paired with habitat protection (*n* = 165) and sign boards (*n* = 154). Habitat protection also commonly co‐occurred with community engagement (*n* = 38) and rescue centres (*n* = 47). However, some actions—particularly community engagement, rescue centres, and sheds—were often suggested independently of others (Figure 5B).

The undirected, weighted network of co‐recommended conservation actions exhibited a significant modular structure (*Q* = 0.27, *p* < 0.0001), indicating the non‐randomness of the clustering of certain conservation measures proposed in FGDs.

**FIGURE 6:**
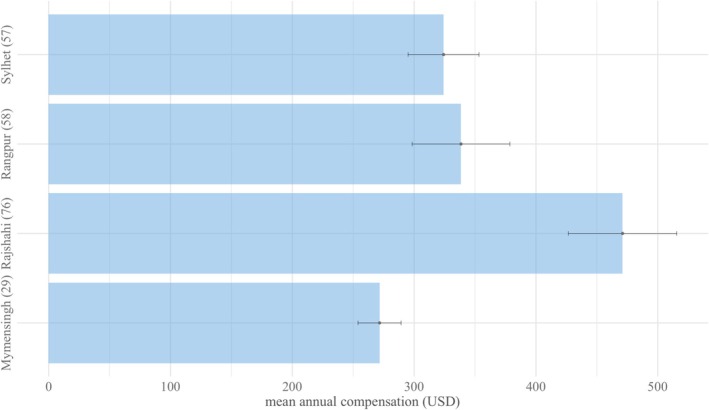
Annual expectations related to 211 heronry sites across four administrative divisions in northern Bangladesh, where landowners cited compensation expectation. Grey line represents standard error. Compensation values were originally reported in Bangladeshi Taka (BDT) and converted to USD using an exchange rate of 1 USD = 122 BDT.

Most FGD sessions (*n* = 211) supported financial compensation to offset perceived losses caused by heronries. However, at 24 sites, landowners cited no compensation expectation. At 211 sites, estimated annual compensation demands varied widely (0–250,000 BDT), averaging USD 348.0 ± 311.6 SD. Compensation expectations also differed across divisions (Figure 6). Mean annual compensation demands were highest in Rajshahi (USD 471.09 ± 387.68 SD), followed by Rangpur (338.46 ± 305.49), Sylhet (324.13 ± 219.76), and Mymensingh (271.62 ± 95.30).

## 4 Discussion

Our findings collectively demonstrate that human‐heronry interactions, both tolerant and antagonistic attitudes, in Bangladesh operate within a broader socio‐ecological system in which ecological outcomes—colony persistence, species composition, breeding success—are mediated by social dynamics at the landowner level. Consistent with SES theory (Ostrom 2009), the persistence of heronries in human‐dominated landscapes is not determined by habitat quality alone, but by the intersection of landowner motivations, experienced costs, perceived threats, and governance structures. Critically, these results challenge the assumption that conservation of Least Concern species in anthropogenic landscapes is straightforward—even species facing no immediate global extinction risk can require complex, locally tailored social interventions to persist in shared wetland‐agricultural landscapes, where human population density averages 1319 people per sq. km (Bangladesh Bureau of Statistics [BBS] 2023).

### 4.1 Human‐Induced Threats

Habitat loss and poisoning emerged as the most severe pressure on heronries in the study area (Figure 2). The pattern is consistent with globally and regionally acknowledged threats to heronry conservation. Nesting tree removal and direct persecution have repeatedly been shown to compromise colony stability and reproductive success (Kushlan 1986; Kushlan and Hafner 2000; Erwin et al. 2003; Naher 2014; Roshnath and Sashikumar 2019; BirdLife International 2024; Byju et al. 2025). For example, Sadik et al. (2025) documented 80 heronry sites abandoned due to habitat loss and direct human disturbance. A similar pattern was observed in Tamil Nadu, India, as Byju et al. (2025) observed a 90.5% decline in heronry nests from 2016 to 2023.

Site‐specific management strategies are therefore essential, as threat profiles varied markedly across divisions (Figures 1 and 2). The prominence of poisoning underscores the localised nature of bushmeat consumption practices (see Datta 2021). For instance, the issue is acute in Sylhet division. This administrative unit, in addition to heronry sites, hosts Ramsar Sites positioned along the East Asian‐Australasian Flyway and receives winter influxes of migratory waterfowls (BirdLife International 2024). In these landscapes, the use of poisoned baits is already an identified threat (Siddique 2023a, 2023b; Hasnat 2023). Poisoning threatens heronry social structures and population dynamics (Datta 2021; Roshnath and Sashikumar 2019) as well as poses a human health concern (see Ibáñez‐Pernía et al. 2022). Stealing and hunting, although perceived as less severe by landowners during FGDs, represent additional pressures exacerbating population declines (Datta 2021), as heronries share human‐use areas where disturbance levels are already high (Naher 2014; Roshnath and Sinu 2023).

### 4.2 Landowners' Tolerance and Motivations

Landowner's tolerance of heronries appears multifactorial, with ethical and environmental considerations at the core, reinforced by social and cultural factors (Figure 3B). The most cited motivations, humane concern, environmental benefits, and social recognition, reflect that conservation support is intertwined with local norms and perceptions. These findings resonate with prior studies from India, where cultural beliefs strongly shaped attitudes toward heronries (see Roshnath 2014; Roshnath and Sinu 2023). In contrast, conditional tolerance—capturing landowners who host heronries despite preferring their removal—exhibited minimal integration with other motivations (Figure 3B), consistent with its conceptually distinct status as a negative motivation rather than an affirmative reason for coexistence, and signalling a latent source of conflict that targeted engagement strategies should prioritise. Addressing multifactorial motivations requires adaptive conservation strategies rather than unilateral approaches, leveraging ethical, environmental, and social incentives simultaneously.

### 4.3 Human‐Heronry Issues

Guano fouling represents the dominant interface of conflict between landowners and heronries in Bangladesh, consistent with patterns documented across colonial waterbird systems globally (Roshnath and Sashikumar 2019; Frank et al. 2021). The strong stench and farming damage reported at most sites (Figure 4A,B) confirm that olfactory and agricultural impacts (e.g., crop contamination, farming damage, and roof fouling)—rather than structural damage or access restriction—are the primary drivers of landowner grievance.

Additionally, most FGDs reported only a single issue—primarily foul smell or farming damage—indicating that these disturbances may operate independently rather than as a cumulative burden for most landowners. In contrast, movement difficulty and roof damage were reported less frequently, suggesting that these issues are more context‐dependent—arising in specific colony configurations such as low‐canopy trees over pathways or nests positioned above structures. Similarly, the network analysis implies that the co‐occurrence of certain issues is predictable, possibly reflecting shared physical attributes of colony sites that simultaneously predispose landowners to multiple problems. For instance, dense roosts may create both odour and obstructed access beneath trees, leading to a paired association of strong stench and movement difficulty.

Despite these reported concerns, we found no evidence of stigma associated with heronry sites, and overall tolerance among landowners remains high (Table 1; Figures 3B and 4B). However, tolerance is not static. Even in communities currently supportive of heronries, persistent and unaddressed issues may override goodwill over time, gradually shifting perceptions from acceptance to frustration. From a management perspective, the observed clustering of problems is useful because it enables targeted mitigation rather than broad, unfocused interventions—a key element for sustaining long‐term coexistence in shared spaces.

### 4.4 Perceived Conservation Measures

Our study highlights that landowners favour habitat protection, awareness programmes, and signage. The clustered pattern suggests a preference for non‐invasive, knowledge‐based, synergistic interventions. Roshnath and Sashikumar (2019) provided similar recommendations for heronry conservation practices. The study suggested that awareness and community engagement programmes combined with habitat protection and monitoring may enhance both compliance and ecological outcomes.

The modular structure in the suggested measures (Figure 5B) indicates that conservation interventions need to be multi‐pronged and context specific. For example, addressing strong stench complaints might be paired with awareness campaigns on ecological benefits and constructing low‐cost tarpaulin sheets under nesting trees as ‘heronry guards’ to minimise guano fouling (see Roshnath and Sashikumar 2019). In parallel, habitat protection could be combined with legal recognition and community engagement to promote conservation stewardship. Building rescue centres for heronries paired with compensation schemes for possible economic loss due to fouling and damage can keep the tolerance level stable. Such integrative nature of the human‐associated problems and perceived measures provides evidence for interdependence between ecological sustainability and social acceptability.

The strong support for financial compensation (Figure 6) reflects an economic dimension of conservation that cannot be overlooked. Incentive‐based programmes have successfully improved community compliance across the Global South (Berkes 2004; Meffe and Carroll 1997). The mean annual compensation expectation of USD 348 ± 311.6 per heronry site represents approximately 47% of the mean annual rural per capita income in Bangladesh (USD 731; BBS 2023), and is roughly equivalent to 60 days of agricultural daily wage labour (BBS 2023). The substantial standard deviation—nearly as large as the mean itself—reflects considerable heterogeneity in landowner expectations across sites, likely driven by differences in colony size, land productivity, and the perceived costs of hosting heronries.

In Bangladesh, colonial waterbirds are protected in the Bangladesh Wildlife (Conservation and Security) Act, 2012. The identification and conservation importance of heronry sites are now recognised, and pellet guns—a common hunting instrument—are banned (Aziz 2023). However, no compensation schemes are currently paired with legal frameworks. Integrating legal frameworks such as community‐based conservation areas or OECM (Other Effective Area‐based Conservation Measures; UNEP‐WCMC 2023) with compensation schemes where contextually appropriate and carefully managed, incentivisation through bird‐watching tourism may formalise and foster sustainable human‐heronry interactions.

### 4.5 Network Approaches in Conservation Social Science

A key methodological contribution of this study is the application of weighted, undirected network analysis to FGD data at a scale—235 sites—rarely achieved in conservation social science. While network approaches have been extensively applied in ecological research, including food webs, species interaction networks, and landscape connectivity, their application to structured social survey data in conservation contexts remains limited (Barnes et al. 2017; Guerrero et al. 2020). Our analysis demonstrates that motivations, problems, and conservation preferences are not randomly distributed but cluster into non‐random modular structures. This has direct implications for conservation planning: targeting one node within a motivational or preference cluster may produce cascading reinforcement across related nodes.

For instance, the pairing of habitat protection and awareness programmes suggests that delivering education campaigns alongside habitat protection initiatives may reinforce both outcomes (Schultz 2011). Similarly, the clustering of strong stench and farming damage as co‐reported problems suggests that on‐site mitigation measures may simultaneously address the most prevalent sources of landowner frustration. Network‐based diagnostic approaches are readily transferable to other human–wildlife coexistence contexts: wherever social survey data contain structured co‐occurrence information, network analysis can reveal leverage points that conventional frequency‐based analyses would miss.

## 5 Conclusion

Three transferable principles emerge from this study with relevance beyond Bangladesh. First, landowner tolerance in shared landscapes is multifactorial and structurally coherent. Conservation strategies must therefore engage ethical, environmental, social, and economic motivational pathways simultaneously rather than relying on single‐lever approaches such as compensation or penalty schemes (Miller et al. 2016). Second, perceived threat severity varies even within a single country, underscoring the need for site‐specific adaptive management rather than uniform national policies. Third, network‐based diagnostics of social survey data offer a scalable, analytically rigorous tool for identifying intervention leverage points—an approach applicable across human‐wildlife coexistence contexts globally.

We acknowledge that reliance on verbal FGD reporting may introduce social desirability bias, and that our cross‐sectional design cannot assess whether current tolerance levels will remain stable over time. Additionally, we excluded individual‐level sociodemographic data—including gender, age, occupation, and educational level—in the analyses, which constrained our ability to assess whether these attributes moderate landowner tolerance, threat perceptions, or conservation preferences. This study, thus, acts as a foundation for more detailed investigations linking ecological outcomes with social interventions. Future studies on longitudinal monitoring could assess whether conservation awareness campaigns, habitat protection measures, or compensation schemes translate into measurable increases in colony persistence and breeding success. Systematic research could incorporate experimental interventions to evaluate the effectiveness of proposed conservation measures. Furthermore, understanding the role of younger generations, gendered perspectives, and migratory waterbird dynamics may refine targeted interventions.

Heronries in Bangladesh persist at the intersection of wetlands' ecological vulnerability and anthropogenic dependence. In heronry conservation, habitat protection emerges as a universal priority, yet it must be operationalised in ways compatible with landowner livelihoods and local land‐use practices. Integrating conservation recognition, community‐level education, on‐site mitigation measures and targeted compensation policy offers a pragmatic pathway for sustaining these critical colonial waterbird populations. Ultimately, translating these insights into broader wetland management policies—guiding human land uses and behaviours toward compatibility with the ecological needs of colonial waterbirds—will be critical for conserving heronries in densely populated agricultural‐wetland landscapes.

## Author Contributions

**Allama Shibli Sadik:** conceptualization (lead), funding acquisition (lead), investigation (lead), methodology (supporting), project administration (lead), writing – original draft (supporting), writing – review and editing (supporting). **Muntasir Akash:** conceptualization (equal), data curation (lead), formal analysis (lead), methodology (equal), visualization (lead), writing – original draft (lead), writing – review and editing (equal). **Ashis Kumar Datta:** conceptualization (equal), investigation (equal), methodology (supporting), visualization (supporting), writing – original draft (supporting), writing – review and editing (lead). **Samiul Mohsanin:** investigation (supporting), writing – review and editing (supporting). **Md. Sher‐E‐Afgan:** investigation (supporting), writing – review and editing (supporting). **Rima Akter:** investigation (supporting), writing – review and editing (supporting). **Humayun Kabir:** investigation (supporting), writing – review and editing (supporting).

## Funding

This work was supported by Sustainable Forests and Livelihoods project Small Innovation Grant, Forest Department, Bangladesh (Ban/SUFAL/IGM/17/2020/1627).

## Disclosure

This study was conceived, designed, and led entirely by researchers based in Bangladesh, with all fieldwork conducted by Bangladeshi researchers and field coordinators across northern and northeastern Bangladesh. The study was embedded within an ongoing national colonial waterbird monitoring programme—the Status, Distribution and Conservation of Colonial Waterbirds in Bangladesh Project—managed by the Bangladesh Forest Department, ensuring direct institutional ownership of the research agenda by a national government body. Analysis and draft preparation were led by a researcher based at the University of Dhaka. Fieldwork and planning were led by co‐authors affiliated with Jahangirnagar University. Local landowners and community stakeholders were not passive subjects of this research but active participants whose knowledge, perceptions, and priorities shaped the four research questions addressed. Findings have been communicated directly to relevant national stakeholders including the Bangladesh Forest Department and Wildlife Conservation Society Bangladesh, both of which are co‐authors on this study, facilitating direct translation of research outcomes into national conservation policy and management practice. All authors are affiliated with institutions in Bangladesh, ensuring that intellectual leadership, data ownership, and publication credit remain within the region where the study was conducted.

## Ethics Statement

The survey was carried out as per the guidelines stated and approved in the Innovation Grant Agreement (Innovation Grant Memo No: Ban/SUFAL/IGM/17/2020/1627) set out by the Sustainable Forests and Livelihoods project Small Innovation Grant Committee of the Forest Department, Bangladesh.

## Conflicts of Interest

The authors declare no conflicts of interest.

## Supporting information


**Table S1:** Focus group discussion questionnaire form used at the heronry sites in northern Bangladesh.

## Data Availability

All files (CSVs, R scripts, RProj, and RData) used to carry out the analyses associated with this manuscript are available at: https://github.com/lynx025/Colonial‐Waterbirds.git.
