# Supplementary material for: Landowner Perceptions of Heronry Conservation in Human‐Dominated Wetlands of Bangladesh
Source: Ecol Evol. 2026 Jul 1;16(7):e73938. doi: 10.1002/ece3.73938 (PMC13320628; doi:10.1002/ece3.73938)
Supplement: Supplementary file 1 — Table S1: Focus group discussion questionnaire form used at the heronry sites in northern Bangladesh. [file ECE3-16-e73938-s001.docx]

Supplementary Material

Table 1. Focus Group Discussion questionnaire form used at the heronry sites in northern Bangladesh

| **Datasheet for Colonial Waterbirds in Bangladesh Night roost/Breeding colony**  (Please give a tick mark/numeric/description in appropriate boxes) | | | | | | | | | | | | |
| --- | --- | --- | --- | --- | --- | --- | --- | --- | --- | --- | --- | --- |
| **Site/location/colony no: Datasheet no:** | | | | | | | | | | | | |
| **Site Info** | | | | | | | | | | | | |
| Date (d/m/y):    Time (24hrs): | | Name of the data collector | | Division: District: Upazilla: Union: Village:  House: | | | | GPS Coordinates N: E: | | Land type: | | |
|  |  |  |  |  |  |  |  |  |  | Khash Land | Private | Total Area (Ha) |
|  |  |  |  |  |  |  |  |  |  |  |  |  |
| **Weather condition** | | Sunny | Cloudy | Rainy | Temperature (°C): | | | | Humidity: | | | |
| **Major Habitat Type** | Agriculture (rice) | | Agriculture (mixed) | | Homestead | |  | |  | |  | |
| **Distances (m) to** | River | | Settlement | | Wetland (beel) | | Road | | Agriculture | | Others | |
|  |  | |  | |  | |  | |  | |  | |
| **Land Owner's Info** | | | | | | | | | | | | |
| Land Owner's Name | | | | Occupation | | | Total land area (Ha): | | Age | Phone No. | | |
| **Night Roost** | | | | | | **Yes** | | | **No** | | | |
| Name of tree Species occupied: | | Estimated number of bird/species: | | | | | | | How long the night roost being used? | | Which time of the year? | |
|  |  |  |  |  |  |  |  |  |  |  |  |  |
|  |  |  |  |  |  |  |  |  |  |  |  |  |
| **Breeding Colony** | | | | | | **Yes** | | | **No** | | | |
| **Breeding Waterbird Colony Info** | | | | | | | | | | | | |
| Tree nesting colony | | | | Yes | No | Ground nesting colony | | | | | Yes | No |
| How long the colony is using the same trees/place? | | | | | | | | Year/s: | | | | |
| Total number of occupied nests / species 1 (…………………………………………………….) | | | |  | | Total number of unoccupied nests/ species 1  (……………………………………………….) | | | | |  | |
| Total number of chicks / *species 1* (…………………………………………………….) | | | |  | | Total number of fledglings / *species 1*  (……………………………………………….) | | | | |  | |
| Total number of occupied nests / species 2 (…………………………………………………….) | | | |  | | Total number of unoccupied nests/ species 2  (……………………………………………….) | | | | |  | |
| Total number of chicks / *species 2* (…………………………………………………….) | | | |  | | Total number of fledglings / *species 2*  (……………………………………………….) | | | | |  | |
| Total number of occupied nests / *species 3* (…………………………………………………….) | | | |  | | Total number of unoccupied nests/ *species 3*  (……………………………………………….) | | | | |  | |
| Total number of chicks / *species 3* (…………………………………………………….) | | | |  | | Total number of fledglings / *species 3*  (……………………………………………….) | | | | |  | |
| Total number of occupied nests / *species 4* (…………………………………………………….) | | | |  | | Total number of unoccupied nests/ *species 4*  (……………………………………………….) | | | | |  | |
| Total number of chicks / *species 4* (…………………………………………………….) | | | |  | | Total number of fledglings / *species 4*  (……………………………………………….) | | | | |  | |
| Total number of occupied nests / *species 5* (…………………………………………………….) | | | |  | | Total number of unoccupied nests/ *species 5*  (……………………………………………….) | | | | |  | |
| Total number of chicks / *species 5* (…………………………………………………….) | | | |  | | Total number of fledglings / *species 5*  (……………………………………………….) | | | | |  | |
| Active breeding months (Total no. of occupied nests per month) | | | | | | | | | | | | |
| Species Name | Jan | Feb | Mar | Apr | May | Jun | Jul | Aug | Sep | Oct | Nov | Dec |
|  |  |  |  |  |  |  |  |  |  |  |  |  |
|  |  |  |  |  |  |  |  |  |  |  |  |  |
|  |  |  |  |  |  |  |  |  |  |  |  |  |
|  |  |  |  |  |  |  |  |  |  |  |  |  |
|  |  |  |  |  |  |  |  |  |  |  |  |  |
|  |  |  |  |  |  |  |  |  |  |  |  |  |
| **Nesting Tree info** | | | | | | | | | | | | |
| Name of the tree species used for nesting | | | Number of trees used for nesting | | Number of total nests | | Distance from human household (Meter) | | Average height of the nests from ground (Meter) | | Distance from Foraging Area to nesting tree | |
| a) | | | a) | | a) | | a) | | a) | | a) | |
| b) | | | b) | | b) | | b) | | b) | | b) | |
| c) | | | c) | | c) | | c) | | c) | | c) | |
| d) | | | d) | | d) | | d) | | d) | | d) | |
| **Direct threats to the waterbird colony** | | | | | | | | | | | | |
| *Anthropogenic threats* | | | *Natural threats* | | | | | *Other threats (Please specify)* | | | | |
| Tree cutting |  | | Tropical storm | | |  | |  | | | | |
| Nest stealing |  | | Predation | | |  | |  | | | | |
| Egg stealing |  | | Nest abandoned | | |  | |  | | | | |
| Chicks stealing |  | | Disease | | |  | |  | | | | |
| Adult got hunted |  | | Chicks fall from the nest | | |  | |  | | | | |
| Poisoned |  | | Lack of fish nearby | | |  | |  | | | | |
| **Landowners’ perception about the waterbird colony** | | | | | | | | | | | | |
| a) Should protect them as they are good for environment | | |  | d) Want to conserve them as they are protected by the wildlife act, 2012 | | | |  | g) what kind of support do you need to protect this location? | | |  |
| b) Protecting them gives social recognition | | |  | e) Don't like their noise and will cut the trees soon | | | |  |  | | |  |
| c) Want to protect them for personal recreation | | |  | f) Don't like them as they litter a lot | | | |  |  | | |  |
| **Problems associated with the waterbird colony** | | | | | | | | | | | | |
| a) | | | | | | | | | | | | |
| b) | | | | | | | | | | | | |
| c) | | | | | | | | | | | | |
| d) | | | | | | | | | | | | |
| e) | | | | | | | | | | | | |
| **Proposed conservation measures** | | | | | | | | | | | | |
| a) | | | | | | | | | | | | |
| b) | | | | | | | | | | | | |
| c) | | | | | | | | | | | | |
| d) | | | | | | | | | | | | |
| e) | | | | | | | | | | | | |
| **Mode of detecting waterbird colony** | | | | | | | | | | | | |
| FD records |  | Social Media | |  | Newspaper | |  | Birding community | |  | | |
| FGD at UP |  | Friend from the locality | |  | Personal communication | |  | Local information | |  | | |
